# The genetic and phenotypic landscapes of Usher syndrome: from disease mechanisms to a new classification

**DOI:** 10.1007/s00439-022-02448-7

**Published:** 2022-03-30

**Authors:** Sedigheh Delmaghani, Aziz El-Amraoui

**Affiliations:** Institut Pasteur, Institut de l’Audition, Université Paris Cité, INSERM UMRS1120, Progressive Sensory Disorders, Pathophysiology and Therapy Unit, F-75012 Paris, France

## Abstract

Usher syndrome (USH) is the most common cause of deaf–blindness in humans, with a prevalence of about 1/10,000 (~ 400,000 people worldwide). Cochlear implants are currently used to reduce the burden of hearing loss in severe-to-profoundly deaf patients, but many promising treatments including gene, cell, and drug therapies to restore the native function of the inner ear and retinal sensory cells are under investigation. The traditional clinical classification of Usher syndrome defines three major subtypes—USH1, 2 and 3—according to hearing loss severity and onset, the presence or absence of vestibular dysfunction, and age at onset of retinitis pigmentosa. Pathogenic variants of nine USH genes have been initially reported: *MYO7A, USH1C, PCDH15, CDH23*, and *USH1G* for USH1, *USH2A, ADGRV1,* and *WHRN* for USH2, and *CLRN1* for USH3. Based on the co-occurrence of hearing and vision deficits, the list of USH genes has been extended to few other genes, but with limited supporting information. A consensus on combined criteria for Usher syndrome is crucial for the development of accurate diagnosis and to improve patient management. In recent years, a wealth of information has been obtained concerning the properties of the Usher proteins, related molecular networks, potential genotype–phenotype correlations, and the pathogenic mechanisms underlying the impairment or loss of hearing, balance and vision. The advent of precision medicine calls for a clear and more precise diagnosis of Usher syndrome, exploiting all the existing data to develop a combined clinical/genetic/network/functional classification for Usher syndrome.

## Introduction

Usher syndrome (USH) is the most common hereditary form of deaf–blindness, with a global prevalence of 4 to 17 cases per 100,000 individuals; it accounts for more than half of all hereditary cases of deaf–blindness and 3–6% of all cases of childhood hearing loss (Hope et al. 1997; Kimberling et al. 2010). This syndrome was first described by Albrecht von Graefe in 1858, but was named after Charles Usher, a Scottish ophthalmologist, who was the first to report its hereditary nature (Bonnet and El-Amraoui 2012; El-Amraoui and Petit 2005; Friedman et al. 2011; Kremer et al. 2006). It displays autosomal recessive inheritance and is clinically characterized by the combination of sensorineural hearing loss (SNHL) and rod–cone dystrophy or retinitis pigmentosa (RP), and variable vestibular dysfunction (Castiglione and Moller 2022; El-Amraoui and Petit 2014; Geleoc and El-Amraoui 2020; Kremer et al. 2006; Mathur and Yang 2015; Nisenbaum et al. 2021).

Usher syndrome is heterogeneous, both clinically and genetically, and has been classified into three distinct clinical subtypes—USH1, USH2, and USH3—based on symptom severity, progression, and age at onset. These three clinical subtypes are associated with nine causal genes encoding various proteins expressed in the inner ear and retina and playing key roles in auditory sensory cell development and function, and photoreceptor maintenance. However, a growing list of additional genes have been reported to be associated with a poorly defined clinical subtype called “atypical Usher syndrome” that does not meet the canonical criteria for the three recognized Usher syndrome subtypes (Bolz 2020; Castiglione and Moller 2022; Fuster-Garcia et al. 2019; Nisenbaum et al. 2021; Nolen et al. 2020; Stiff et al. 2020). Moreover, for each Usher syndrome-associated gene, clinical features can vary according to the type and location of the causal gene, making it difficult to achieve an accurate diagnosis and classification of Usher syndrome (Bolz 2020; Castiglione and Moller 2022; Fuster-Garcia et al. 2019; Nisenbaum et al. 2021; Nolen et al. 2020; Stiff et al. 2020). The early and accurate diagnosis of Usher syndrome is essential for effective clinical management of the patient, and to improve our understanding of genotype–phenotype relationships in USH, in anticipation of treatment options, including cochlear implantation. Here, we summarize key information about the clinical and genetic aspects of Usher syndrome. The list of genes associated with Usher syndrome has grown in recent years. We therefore revisit this list, considering the available information about the classic Usher genes and the atypical and ultra-rare Usher-like forms, and the function of the corresponding proteins in the inner ear and eye. The redefinition of criteria for Usher syndrome is important for clinicians, scientists, and patients; it will improve patient screening and counseling, and accurately guide the development of precision medicine-based therapies.

### Anatomy and function of the inner ear and eye, the sensory organs targeted by Usher syndrome

The main target organs of Usher syndrome are the inner ear and eye. The extent of the sensory deficit in these two sensory organs determines the severity of clinical symptoms, according to the form of Usher syndrome. Below, we provide general information about the anatomy of these organs, focusing on key Usher target cells.

## The inner ear, and the sensory hair cells

**Fig. 1:**
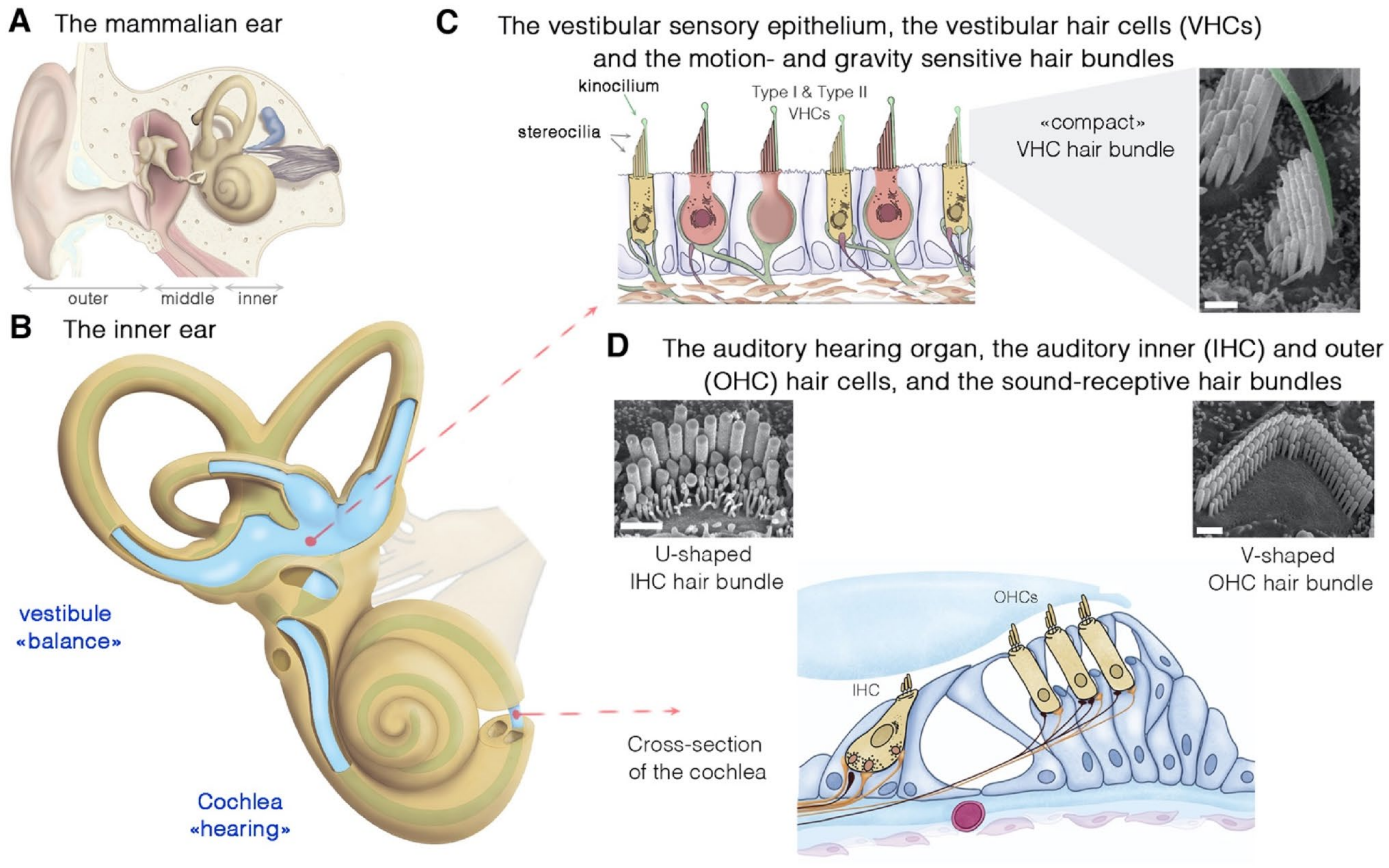
Anatomy of the mammalian ear, and the balance and hearing sensory organs in the inner ear. **A** The mammalian ear consists of 3 compartments: the outer, middle and inner ear. **B** The inner ear contains the vestibule (balance organ), and the cochlea (hearing organ). **C**,** D** The vestibular (**C**) and auditory (**D**) sensory epithelia are composed of the hair cells, associated innervation, and various types of supporting cells. In the vestibule, type I and type II vestibular hair cells (VHCs) sense head and body motion. In the cochlea, hearing results from the processing of sound waves by the hair cells, which are of two types: the inner hair cells (IHCs), which are the genuine auditory sensory cells responsible for signaling to the brain, and the outer hair cells (OHCs), which act as mechanical amplifiers, conferring on the mammalian cochlea its high sensitivity, and frequency discrimination capacity (low frequencies at the apex (20 Hz)-high frequencies at the base (20 kHz) in humans). The scanning electron micrographs illustrate the staircase pattern of the highly organized hair bundles, arranged in different shapes in different types of hair cell (**C**,** D**). In mammals, only the vestibular hair bundle retains the kinocilium (artificially colored in green in **C**), a true cilium that stands at the tallest edge of the hair bundle. Scale bar = 1 μm

The inner ear houses the sensory organ for balance, the vestibule, and the organ responsible for hearing, the cochlea (Fig. 1A, B) (Delmaghani and El-Amraoui 2020). Balance and hearing depend on the process of mechanoelectrical transduction, which occurs in highly specialized polarized epithelial cells called hair cells: type I and type II hair cells in the vestibule (vestibular hair cells or VHCs) (Fig. 1C), and the inner (IHC) and outer (OHC) hair cells in the cochlea (Fig. 1D). The mechanosensitive organelle in all hair cells is the hair bundle (Delmaghani and El-Amraoui 2020). It consists of 50–100 F-actin-filled stereocilia that project a few micrometers above the apical cell surface, forming a staircase pattern increasing towards a single transient genuine cilium, the kinocilium (which is only transient during the development of cochlear sensory cells) (Fig. 1C, D) (Delmaghani and El-Amraoui 2020).

The basic principles of hair-bundle function are similar between the vestibule and cochlea, but hair-bundle organization, geometry, physical and functional properties vary according to the position of the hair cell in the corresponding sensory organ. Vestibular hair bundles are often compact, with a staircase composed of four to five rows of stereocilia. In the cochlea, the OHC hair bundles are arranged in three rows, forming a distinct "V"-shaped (or W-shaped) staircase, whereas the IHC hair bundles have a flatter, slightly curved profile, forming a U-shape (Fig. 1C, D). The cochlear hair bundles are extremely sensitive; they convert nanometer displacements of the stereocilia induced by sound waves into changes in membrane potential. In the IHCs, this results in neurotransmitter release, and signal transmission to the brain (Fig. 1; see (Delmaghani and El-Amraoui 2020)).

## The retina, and the sensory photoreceptor cells

**Fig. 2:**
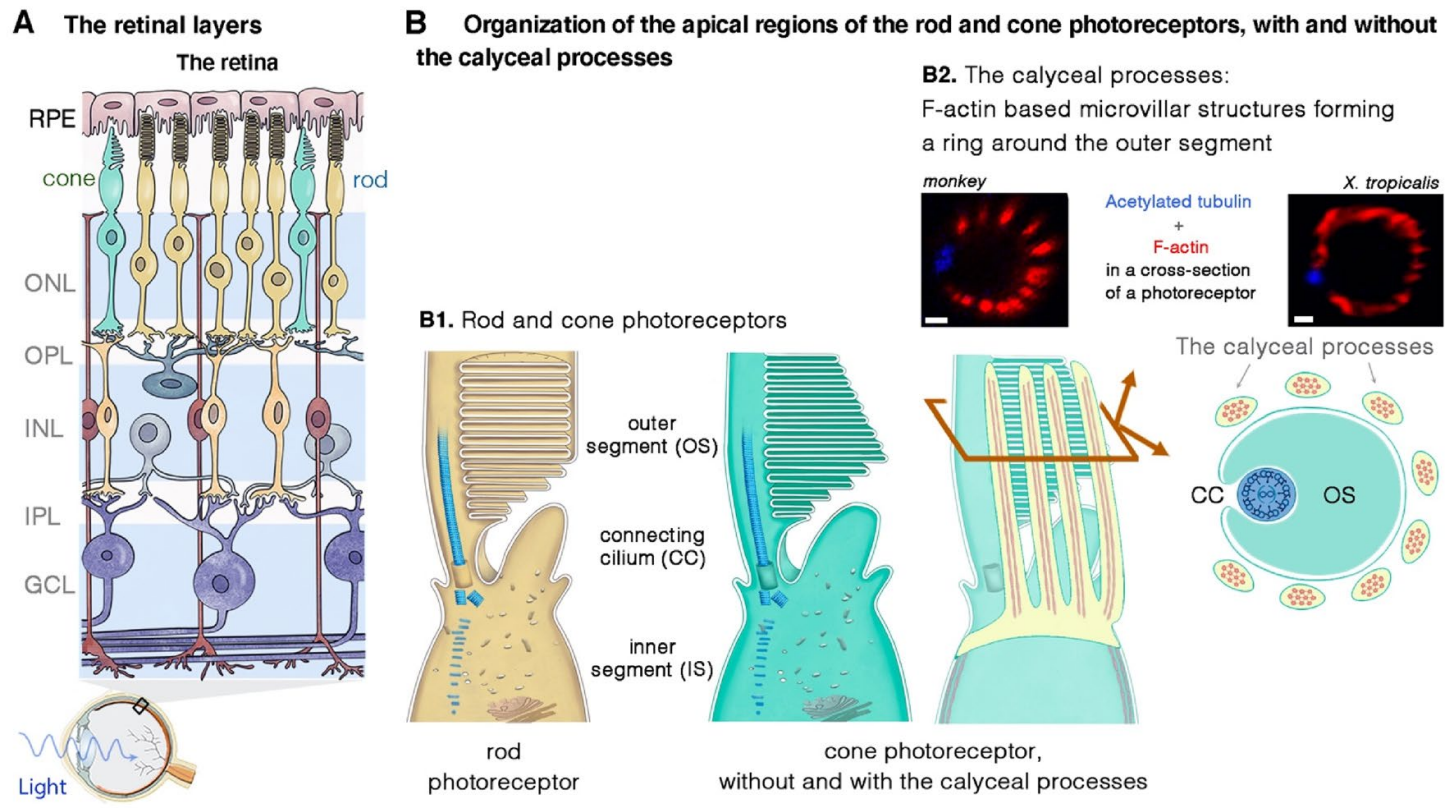
Anatomy of the eye, the retinal layers and the light-sensitive rod and cone photoreceptor cells. **A** The retina is a neural layer lining the back of the eye and containing retinal pigment epithelium cells (RPE) together with rod and cone photoreceptor cells. The photoreceptors are connected to various horizontal cells, bipolar cells, amacrine cells and ganglion cells present in the inner retina. Müller cells are glial cells that span all cell layers of the retina. **B** Schematic diagram illustrating the apical region of the photoreceptor cells. **B1.** Despite differences in their morphological architecture, the rod and cone photoreceptor cells have the same apical organization: a connecting cilium with an axoneme connects the light-sensitive outer segment consisting of tightly packed membrane disks with the inner segment containing all the organelles required for energy and protein synthesis. The connecting cilium and the periciliary ridge region form the periciliary membrane complex. **B2.** In some species (e.g., amphibians, non-human primates and primates), microvillus-like structures emerge from the top of the inner segment and form a collar around the base of the photoreceptor outer segment. These finger-like structures, visible on the scanning electron micrograph, are known as calyceal processes. Scale bar = 1 μm. ONL, outer nuclear layer; INL, inner nuclear layer, GCL, ganglion cell layer; OPL, outer plexiform layer; and INL, inner plexiform layer

In the eye, light must pass through several structures and compartments—the cornea, lens, vitreous, and retinal cell layers—before it reaches the photoreceptor cells (rods and cones) (Botto et al. 2021; Crane et al. 2021). The visual signal is initiated by the photosensitive opsins, which trigger a phototransduction cascade in the outer segment (the site of phototransduction) of the photoreceptor cells (Fig. 2A, B). The outer segment contains hundreds of membrane disks stacked into an ordered array, organized around a microtubule-based axoneme that begins in the distal portion of the inner segment and passes through the connecting cilium extending along the edge of the outer segment base (Fig. 2B1) (Botto et al. 2021; Crane et al. 2021).

**Fig. 3:**
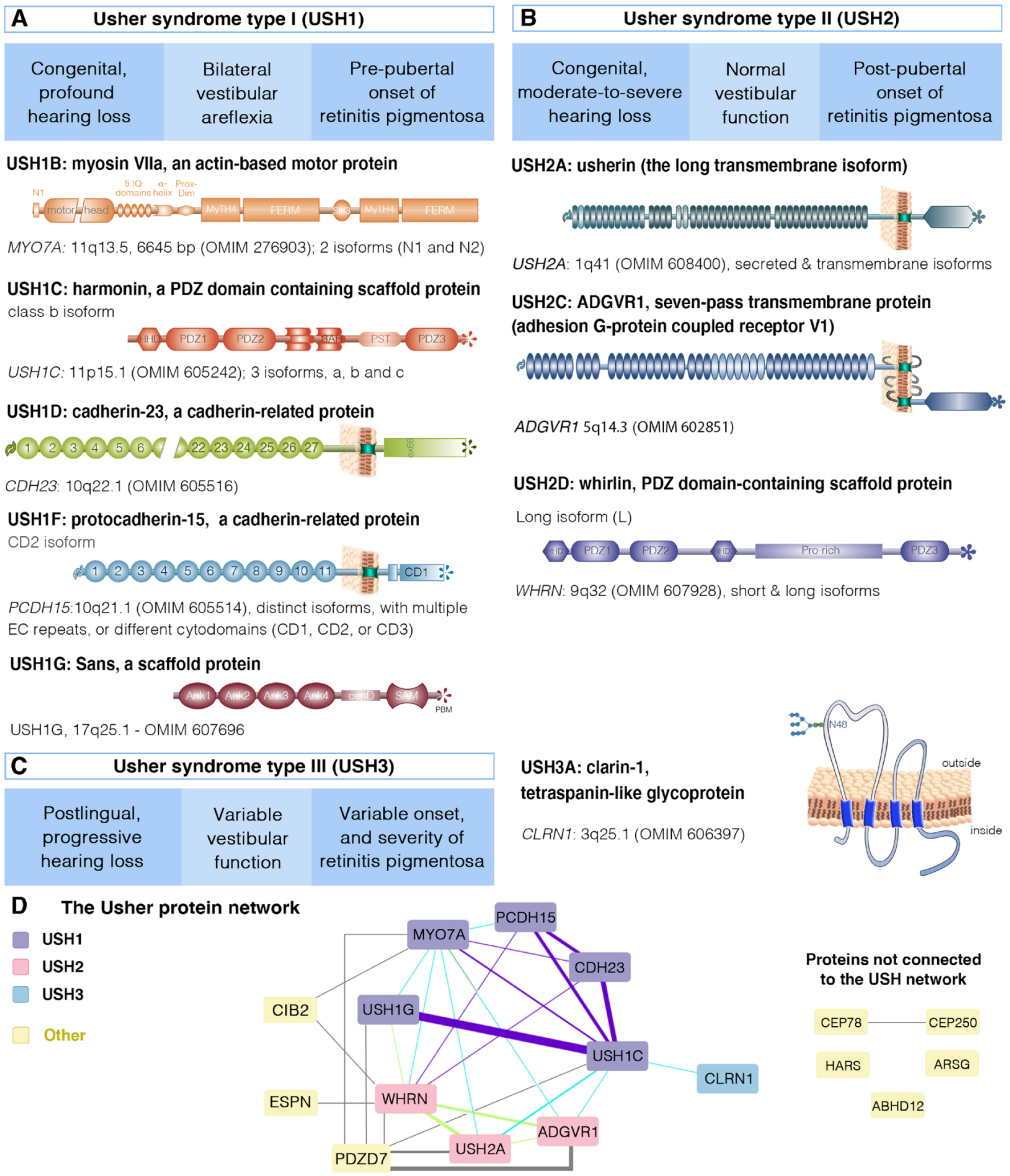
Illustration of the differences between the three clinical forms of Usher syndrome and the Usher proteins. Usher syndrome (USH) is classified into three types—USH1 (**A**), USH2 (**B**) and USH3 (**C**)—on the basis of age at onset and severity of hearing loss, the presence or absence of vestibular deficits, and age at onset of retinitis pigmentosa. Based on genetic evidence and the functions of Usher proteins and phenotypic manifestations in disease models, there is good evidence for nine undisputed Usher syndrome genes: Five USH1 genes **A** *MYO7A* (myosin VIIa, responsible for USH1B), *USH1C* (harmonin, responsible for USH1C), *CDH23* (cadherin-23, responsible for USH1D), *PCDH15* (protocadherin-15, responsible for USH1F); three USH2 genes **B** *USH2A* (usherin, responsible for USH2A), *ADGRV1* (ADGRV1, adhesion G protein-coupled receptor V1, responsible for USH2C), *WHRN* (whirlin, responsible for USH2D), and one USH3 gene **C** *CLRN1* (clarin-1, responsible for USH3A). For more details on any USH gene, please refer to the references for the corresponding OMIM number, and < http://hereditaryhearingloss.org > . **D** The Usher network analysis using Cytoscape software. Among USH1 interactions, almost all interactions (5/7) were identified at least once using detection methods indicative of direct physical contact between proteins (purple edges). The blue, and green lines indicate the existence of experimental evidence for the involvement of these proteins in one 'physical association' or more 'association' complexes, respectively. Among the other proteins referred to as USH molecules (yellow boxes), several has no connection to the classic USH proteins

One key property of the photoreceptor cells is the daily renewal of the membranous disks in the photosensitive outer segment (Young 1967, 1974). The new disks (~ 10% are turned over each day) form at the base of the outer segment, and the old disks are taken up into the retinal pigment epithelium cells surrounding the distal tip of the outer segments by phagocytosis (Young 1974). This daily turnover of the outer segment disks is sustained by the continual generation of phototransduction proteins in the (metabolic) inner segment and their delivery to the OS via the connecting cilium. Every minute, ~ 2000 molecules of opsin, the predominant protein in the disks, pass through the connecting cilium (Besharse and Wetzel 1995; Young 1967); Fig. 3B2). There may be differences between species in terms of the cellular architecture and distribution in the retina of the light-sensitive photoreceptor cells, the rods and cones. For example, the fovea, which underlies high-acuity diurnal vision thanks to its enrichment in cones, is absent from the rodent retina. Furthermore, in some species (e.g., amphibians, non-human primates, and humans, microvillus-like structures called calyceal processes emerge at the tip of the inner segment and surround the base of the outer segments (see (Sahly et al. 2012); Fig. 3B2). However, the presence of two to three apical processes/membrane projections extending along the edge of the outer segment, as observed in mice, does not meet the definition for calyceal processes. Calyceal processes were first described 50 years ago (Brown et al. 1963; Cohen 1963; Ulshafer et al. 1987). They are specialized membrane processes satisfying the following three criteria: (i) they must contain a sufficiently large number of apical processes, generally 8 to 16; (ii) organized in a ring pattern, and forming a calyx around the outer segment, and (iii) labeled by F-actin staining, demonstrating their microvillus-like nature due to the presence of a core of actin filaments (Fig. 3B).

## The classification of Usher syndrome: three typical Usher clinical forms

### Clinical features

Usher syndrome is clinically heterogeneous and characterized by a combination of sensorineural hearing loss (SNHL) and retinitis pigmentosa (RP), with variable vestibular dysfunction. It is traditionally classified into three different subtypes—USH1, USH2, and USH3—depending on age at onset, severity and progression, and the presence or absence of vestibular dysfunction (Hope et al. 1997; Kimberling et al. 2010; Nisenbaum et al. 2021; Toms et al. 2020b) (see Fig. 3A–C).

Usher type 1 (USH1) is the most severe form, accounting for about 25–44% of all cases of Usher syndrome (Bonnet et al. 2016; Fuster-Garcia et al. 2021; Jouret et al. 2019; Toms et al. 2020b). It is characterized by severe-to-profound congenital SNHL, vestibular areflexia, and a prepubertal onset of retinitis pigmentosa (RP) (Kremer et al. 2006; Mathur and Yang 2015; Geleoc and El-Amraoui 2020) (Fig. 3A). Many USH1 patients do not begin to walk before the age of 18 months, and vestibular areflexia is responsible for the delayed motor development observed in these patients (Loundon et al. 2003). The patients compensate for their vestibular areflexia through vision until the onset of RP, although they often present difficulties performing activities that require balance (Loundon et al. 2003). USH1 retinopathy is classified as a rod–cone dystrophy, with rod abnormalities appearing first and rapidly worsening, followed by slowly progressing cone dysfunction, and photoreceptor cell degeneration (Jacobson et al. 2008, 2011; Khateb et al. 2019). Night blindness is the first symptom of RP and may be followed by a narrowing of the field of vision (“tunnel vision”), which rapidly progresses to complete blindness by the age of 30 years (Jacobson et al. 2008) (Jacobson et al. 2011; Khateb et al. 2019).

Usher type 2 (USH2) is the most common form of USH, accounting for more than half of all cases (Bonnet et al. 2016; Jouret et al. 2019; Toms et al. 2020b; Fuster-Garcia et al. 2021). USH2 patients typically have a sloping audiogram, mild-to-moderate congenital SNHL for low frequencies and severe-to-profound SNHL for higher frequencies (Abadie et al. 2012) (Fig. 3B). Speech is not usually affected, and vestibular function is normal. Symptoms of rod–cone dystrophy manifest later in USH2 patients than in USH1 patients, as retinitis pigmentosa is not usually diagnosed until the second decade of life, or later (Van Aarem et al. 1995; Millan et al. 2011; Pierrache et al. 2016; Toms et al. 2020a).

Usher type 3 (USH3) is the least common type of USH, accounting for 2–4% of all cases, but with a high prevalence in Finnish and Ashkenazi Jewish populations, probably due to founder effects (Adato et al. 2002; Ben-Yosef and Friedman 2003; Malm et al. 2011; Millan et al. 2011; Ness et al. 2003; Marouf et al. 2022). The audiovestibular features of USH3 are the most variable among Usher subtypes (Khan et al. 2017; Ness et al. 2003). USH3 patients display progressive, post-lingual SNHL, beginning at higher frequencies, mostly during childhood, although onset may occur as late as the age of 35 years (Ness et al. 2003) (Fig. 3C). Progression is variable but, in most cases, the patients ultimately become profoundly deaf (Adato et al. 2002; Khan et al. 2017; Ness et al. 2003). USH3 patients have well-developed speech. Vestibular dysfunction is variable and occurs in about half the patients (Adato et al. 2002; Ben-Yosef and Friedman 2003; Malm et al. 2011; Millan et al. 2011; Ness et al. 2003). Retinitis pigmentosa also has a variable onset, but is post-pubertal, generally occurring after the age of 20 years (Herrera et al. 2008; Malm et al. 2011).

### The genetic landscape of the Usher syndrome

In addition to its clinical heterogeneity, Usher syndrome is also genetically heterogeneous. Nine genes have been clearly identified as responsible for the three subtypes of Usher syndrome (Fig. 3A–C). USH1 is caused by pathogenic variants of the following five genes: *MYO7A* (myosin VIIA; USH1B) (Weil et al. 1995), *USH1C* (harmonin; USH1C) (Verpy et al. 2000), *CDH23* (cadherin 23; USH1D) (Bork et al. 2001) (Bolz et al. 2001), *PCDH15* (protocadherin 15; USH1F) (Ahmed et al. 2001), and *USH1G* (sans; USH1G) (Weil et al. 2003) (Fig. 3A). USH2 is caused by pathogenic variants of the following three genes: *USH2A* (usherin; USH2A) (van Wijk et al. 2004; Weston et al. 2000), *ADGRV1* (adhesion G protein-coupled receptor V1; USH2C) (Weston et al. 2004), and *WHRN* (whirlin; USH2D) (Ebermann et al. 2007) (Fig. 3B). *CLRN1* (clarin 1; USH3A) is the only gene shown to be associated with USH3 (Adato et al. 2002; Fields et al. 2002) (Fig. 3C).

These USH genes encode proteins from different classes and families with different functions, including motor proteins, scaffold proteins, transmembrane proteins, adhesion proteins, and G protein-coupled receptors (Fig. 3). USH proteins are organized into a protein “interactome” in both the inner ear and the retina, where the Usher molecular complexes have been shown to be crucial for the development and function of the auditory sensory cells and for the maintenance of retinal photoreceptor cells (El-Amraoui and Petit 2005, 2014; Kremer et al. 2006; Maerker et al. 2008; Bonnet and El-Amraoui 2012; Mathur and Yang 2015; Geleoc and El-Amraoui 2020).

## Function of Usher proteins in health and disease

**Fig. 4:**
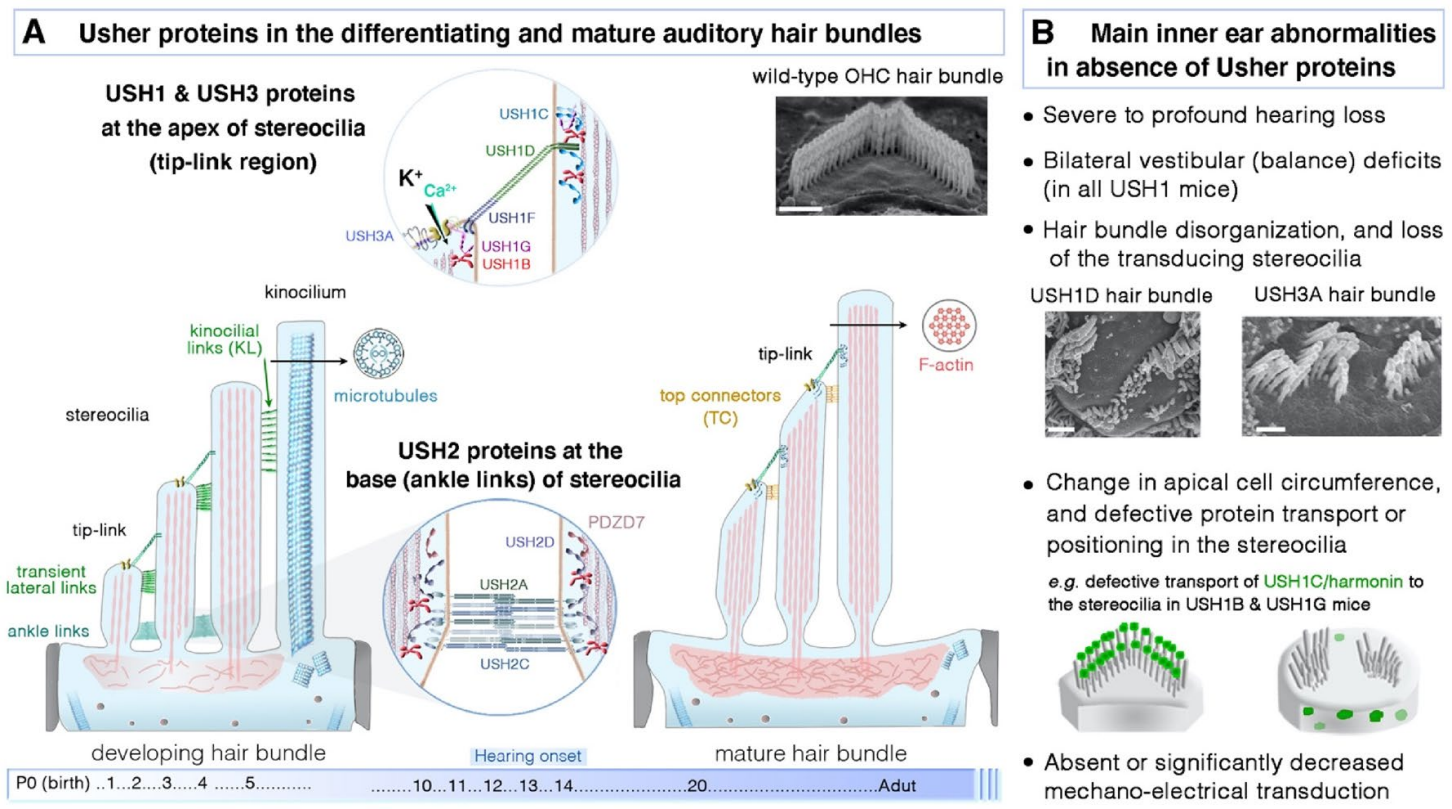
Usher proteins in the hair bundle and the principal related pathogenic phenotypic features in the inner ear. **A** Developing and mature stages of the cochlear hair bundle illustrating the dynamic changes in the various types of hair-bundle link: the transient apical lateral links, ankle links, and kinocilial links disappear after bundle maturation, by P12, in mice. The top connectors develop late in differentiation and persist in the mature hair bundles. The tip link is a unique fibrous link that connects the tip of a stereocilium to the side of the adjacent stereocilium. It is present in both developing and mature hair bundles. In mammals, the kinocilium, with its 9 + 2 axoneme pattern and typical motile cilium structure, is absent from mature hair bundles. The middle close-up views illustrate the function of USH proteins in the hair bundle. Cadherin 23 homodimers and protocadherin 15 homodimers interact to form the tip links, with protocadherin 15 forming the lower component that gates the MET channels. Myosin VIIa, harmonin, and sans cross-link the stereociliary membrane to the core of actin filaments. USH2 proteins are the key components of the ankle links, which eventually disappear and are absent from mature auditory hair bundles. It remains unclear whether USH3A acts as an accessory protein of the MET channel complex. **B** Usher protein defects cause a wide range of physiological, morphological, and molecular abnormalities; the main phenotypic findings for USH1 are listed. The USH3 SEM micrograph is adapted from (Dulon et al. 2018). Scale bar = 1 μm

**Fig. 5:**
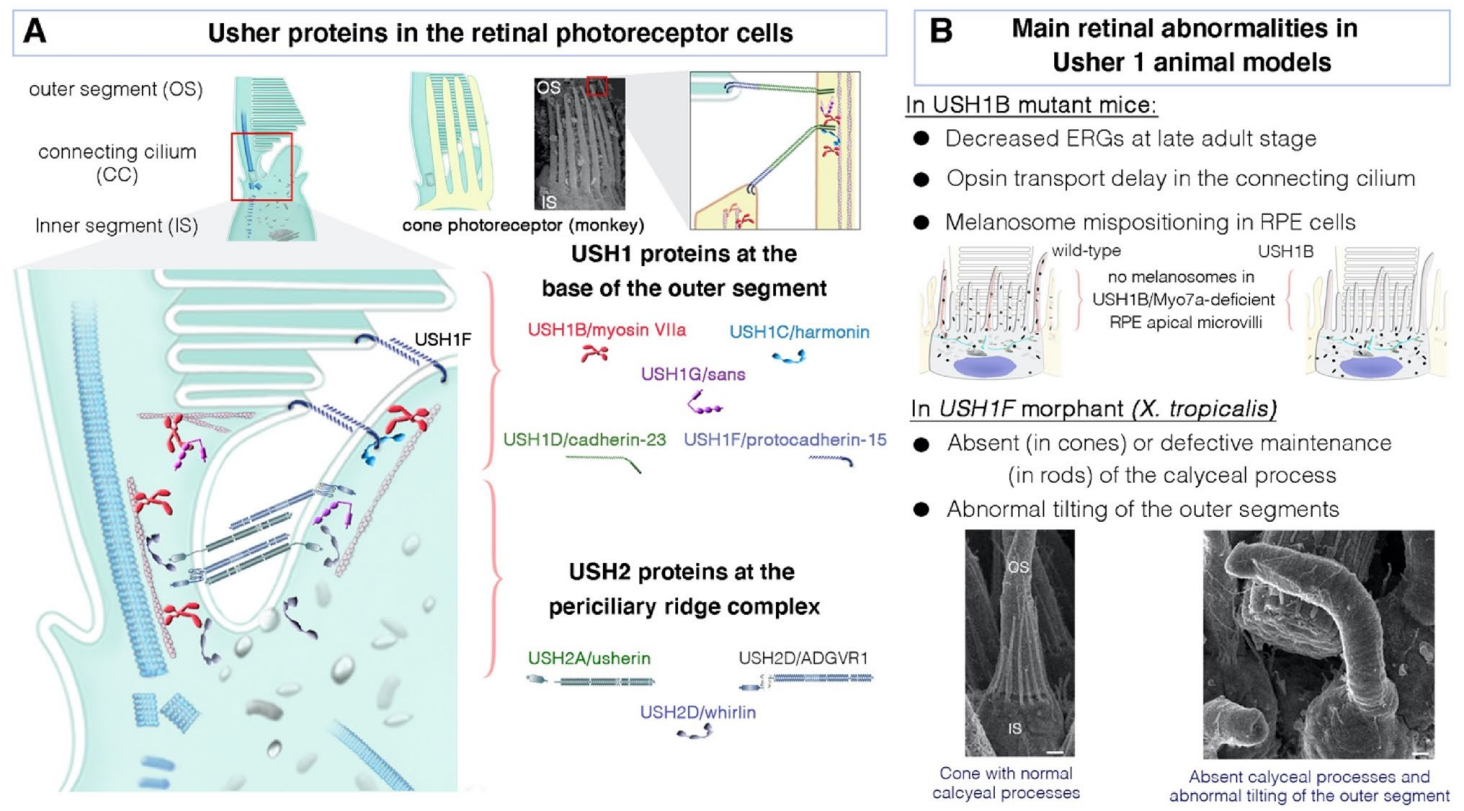
Usher proteins in photoreceptor cells and the principal related pathogenic phenotypic features in the retina. **A** The functional assemblies of Usher proteins operating in the hair bundle also play analogous roles in the photoreceptor cells, notably in the periciliary ridge membrane complex (PMC) and the calyceal processes (when present). Based on their presence close to the vesicle loading point at the base of the PMC region, the USH2 proteins (along with myosin VIIa and sans) were thought to play a role in vesicle transport between the inner and outer segments of the photoreceptors. Recent evidence suggests that USH1 proteins play a key role at the interface between the inner and outer segments, probably controlling the size of newly produced membrane disks and contributing to their correct organization in the apical zone of the PMC region. **B** As in the inner ear, USH protein defects cause diverse phenotypic abnormalities in the retina, including decreased electroretinogram responses (ERGs), probably due to misshapen photoreceptor disks (as shown in *Xenopus* morphants, adapted from Schietroma et al. (2017)). Other abnormalities—opsin transport delay and melanosome mispositioning—are observed in absence of Myo7a. The precise distribution of clarin-1 in the retina is currently unknown, and there are no mouse USH3A models for the retinal phenotype, limiting the accuracy of extrapolations of USH3 function in the eye. Scale bar = 1 μm

The precise sequence of pathogenic events leading to sensory loss (hearing, balance, and vision) in Usher syndrome remains unclear. However, based on the known properties of Usher proteins and elucidation of the molecular complexes they form, steady progress has been made over the years towards understanding how each Usher protein cooperates in well-defined and orchestrated pathways in the inner ear and retinal Usher target cells. Multiple protein–protein interactions have been identified between USH proteins (Adato et al. 2005b; El-Amraoui and Petit 2005, 2014; Kremer et al. 2006; Maerker et al. 2008; Mathur and Yang 2015; Geleoc and El-Amraoui 2020) (see Fig. 3D). Through search of the literature and protein–protein interaction databases, we used Cytoscape (Shannon et al. 2003), considering the IMEx curation rules (Orchard et al. 2012) to reconstitute the Usher network. This reveals that the nine Usher proteins are linked to a total of 20 interactions reported for mouse and human orthologous proteins (see Fig. 3D). The number of connections varies greatly between the 9 Usher proteins (colored boxes), from 1 (CLRN1) to 7 (MYO7A and USH1C), with an average of 4 neighbors per protein (Fig. 3D). Extensive studies of the animal models available for each form of Usher syndrome (reviewed in El-Amraoui and Petit 2005, 2014; Reiners et al. 2006; Kremer et al. 2006; Maerker et al. 2008; Mathur and Yang 2015; Geleoc and El-Amraoui 2020), have shed light on how the lack of a given USH protein can affect the subcellular distribution of at least one other USH protein in hair cells and/or photoreceptor cells, further confirming in vivo the interdependence of USH proteins for normal function (Boeda et al. 2002; Senften et al. 2006; Lefevre et al. 2008). All the available data suggest that the hair cells and photoreceptor cells, which have structural and functional characteristics in common, are the key primary targets of Usher sensory deficits (El-Amraoui and Petit 2005, 2014; Kremer et al. 2006; Reiners et al. 2006; Maerker et al. 2008; Cosgrove and Zallocchi 2014; Mathur and Yang 2015; Geleoc and El-Amraoui 2020). Detailed information about the complete spatiotemporal expression profiles (e.g., in synaptic regions, adherens junctions) of USH proteins in hair cells and photoreceptors, and the additional roles of these proteins in other cells in the retina (e.g., Müller cells, amacrine cells, and RPE cells), the brain and beyond is provided by other recent reviews (Reiners et al. 2006; Maerker et al. 2008; Gregory et al. 2011; El-Amraoui and Petit 2014; Mathur and Yang 2015). We will focus here on holistic molecular working models illustrating, for each form of Usher syndrome, the key contributions of the complexes to the development, differentiation, and functioning of the hair bundle (Fig. 4), and at the interface between the inner and outer segments of photoreceptor cells (Fig. 5).

## USH proteins during morphogenesis of the mechanosensitive hair bundles

Fortunately, mouse inner ears and their specialized mechanosensitive hair cells work much like those of humans (Bowl and Dawson 2015). This has made it possible to make steady progress towards understanding the precise mechanisms underlying the hearing and balance deficits of Usher syndrome (Delmaghani and El-Amraoui 2020). An abnormal shape and dysfunction of the hair bundles is common to all Ush mutant mice, with severity depending on the type of hair cell and genetic type of USH (El-Amraoui and Petit 2005; Petit and Richardson 2009; Bonnet and El-Amraoui 2012; Mathur and Yang 2015; Geleoc and El-Amraoui 2020). During morphogenesis and in the mature hair bundles, the stereocilia and kinocilium (when present) are interconnected by several morphologically and molecularly different fibrous links (Goodyear et al. 2005). In all hair bundles, a single obliquely inclined tip-link (TL) connects the tip of the distal stereocilium to the shaft of the adjacent taller stereocilium (Kachar et al. 2000). Transient links are prominent in developing hair bundles: the transient lateral links (tLL, at the apex of stereocilia), ankle links (AL, located in or just above the tapered region at the base of each stereocilium), and kinocilial links (KL; connecting the kinocilium to the adjacent stereocilia) (Goodyear et al. 2005). Only the tip links and the top connectors just below them persist in mature adult hair bundles (Fig. 4A) (Goodyear et al. 2005). Collectively, protein–protein interaction studies and the characterization of animal models of Usher syndrome have led to the establishment of models explaining the hearing and balance dysfunctions in this syndrome (Bonnet and El-Amraoui 2012; El-Amraoui and Petit 2005; Geleoc and El-Amraoui 2020; Mathur and Yang 2015; Michalski and Petit 2019). For USH1, myosin VIIa and sans are required, in early differentiating hair cells, for the transfer of most USH1 and USH2 proteins from the apical surface of the hair cell to the stereocilia, where they become restricted to the tip region. Once in the stereocilia, cadherin-23 and protocadherin-15 heterodimers form the transient fibrous apical links connecting the stereocilia to each other and to the kinocilium (see Fig. 4A) (Boeda et al. 2002; Siemens et al. 2002). The other three USH1 proteins—harmonin, sans and myosin VIIa—anchor these links to the actin filaments of the stereocilia, probably mediating the tension applied to the early embryonic lateral links anchored to actin filaments and ensuring the cohesion and correct orientation of the hair bundle Fig. 4) (Boeda et al. 2002; Siemens et al. 2002; Lefevre et al. 2008; Michalski et al. 2009; Grillet et al. 2009; Bahloul et al. 2010).

For USH2, the three UHS2 proteins—usherin, ADGVR1 and whirlin—are transiently colocalized at the base of the stereocilia, where they are thought to be components of the ankle-link molecular complex (Adato et al. 2005a; McGee et al. 2006; Michalski et al. 2007; Yagi et al. 2007)}. Myosin VIIa and PDZD7, expressed at the base of the stereocilia, are also components of this complex (Zou et al. 2014). A lack of USH2 proteins affects the formation of ankle links (McGee et al. 2006; Michalski et al. 2007; Yagi et al. 2007). These links are transient, remaining present during development almost up to the final maturation of the hair bundle (Goodyear et al. 2005). Their absence leads to misshaped OHC bundles (McGee et al. 2006) (Michalski et al. 2007; Yagi et al. 2007), more prominently at the base of the cochlea and probably accounting for the severity of hearing loss for high-frequency sounds in USH2 patients. USH1 proteins are produced and normally targeted to the hair bundle in Usher 2 mouse models. This normal presence of USH1 proteins accounts for the cohesive coupling of the stereocilia being maintained during hair-bundle morphogenesis. Conversely, the compact hair bundles of vestibular hair cells may be able to cope with the minimal misshaping caused by the loss of USH2 proteins, accounting for the normal vestibular function in USH2 patients.

For USH3A, a misalignment of the hair bundles, with occasional bundle fragmentation, has been observed in the absence of clarin-1 (Geng et al. 2009; Dulon et al. 2018) but it remains unclear how the absence of clarin-1 leads to such disorganization.

## USH1 and USH3A proteins at the core of the mechanoelectrical transduction machinery

Studies of conditional mutant mice in which USH proteins are inactivated only at postnatal stages have shown that the USH1 and USH3 proteins also play crucial roles in the mature adult hair bundle (Fig. 4). Several studies have shown that, in mutant mice, mechanoelectrical transduction (MET) is altered in the absence of a particular USH1 protein or USH3A, demonstrating an essential role for these proteins in the MET machinery. Cadherin-23 and protocadherin-15 not only contribute to the early transient lateral links, but they also form the upper and the lower tip-link, respectively. This link is thought to gate the mechanoelectrical transduction channels (see Fig. 3). It has been suggested that myosin VIIa, sans and harmonin form the core of the upper tip-link density complex, a multiprotein platform ensuring dynamic crosslinking of the tip-link to the actin filaments of the stereocilia (Caberlotto et al. 2011; Grati and Kachar 2011; Grillet et al. 2009; Michalski et al. 2007). It remains unclear how the absence of clarin-1 disrupts MET currents, and whether this tetraspanin-like glycoprotein acts as an accessory protein of the MET channel complex. One structural phenotype common to all USH1 and USH3A mutant mice is a progressive regression of the mechanotransducing stereocilia (short and middle rows of stereocilia). This observation highlights the positive role of the intact mechanoelectrical transduction machinery and F-actin polymerization in maintaining the correct length of the stereocilia (Caberlotto et al. 2011), as supported by experimental, mechanical and functional investigations (Velez-Ortega et al. 2017).

## USH proteins have key functions at the interface between the inner and outer segments of photoreceptor cells

Age at onset, progression and severity differ between the various forms, but all USH patients ultimately develop retinitis pigmentosa, leading to blindness (El-Amraoui and Petit 2014; Kremer et al. 2006; Mathur and Yang 2015; Geleoc and El-Amraoui 2020; Toms et al. 2020b; Nisenbaum et al. 2021; Castiglione and Moller 2022). By contrast, despite the occurrence of quantifiable structural and functional retinal abnormalities in many Ush mice, only a few USH2 models develop unambiguous retinal cell degeneration and vision defects (Yang et al. 2010). The discrepancy between the mouse and human retinal phenotypes has made it particularly difficult to unravel the precise mechanism of Usher vision loss. Possible explanations for this phenotypic discrepancy include the shorter lifespan of the mouse, differences in light exposure (diurnal versus nocturnal species), the functional redundancy of USH proteins in the retina, and intrinsic structural differences in photoreceptor cell architecture (El-Amraoui and Petit 2005). One key structural difference between species is the presence or absence of well-developed calyceal processes in the retina (Sahly et al. 2012). These microvillus-like structures are present in the photoreceptors of humans, macaques, and frogs, but not in those of mice or other rodents (Sahly et al. 2012). The generation and characterization of novel animal models better mimicking human ocular USH disease have proved crucial for gaining insight into the molecular mechanisms underlying retinal dysfunction and degeneration in USH (Dinculescu et al. 2021; Mathur and Yang 2015, 2019; Williams 2002). As for the USH1 complex in hair bundles, there is increasing evidence for a holistic molecular mechanism involving the five USH1 proteins (myosin VIIa, harmonin, cadherin-23, protocadherin-15 and sans) acting together in photoreceptor cells (Sahly et al. 2012). Studies in amphibian (Schietroma et al. 2017) and zebrafish (Miles et al. 2021) models have suggested that a defect of the USH1 protein network between the photoreceptor outer segment and the calyceal processes is probably the cause of USH1 retinal dystrophy in humans (see Fig. 5). Interestingly, a pig model of USH1C lacking harmonin, *USH1C*^R31X^, was recently reported to mimic the human disease, displaying combined deafness, changes in photoreceptor architecture, and significantly impaired visual function (Grotz et al. 2021).

The use of sophisticated experimental protocols in USH mutant mice, some of which display a late-onset progressive decrease in electroretinogram (ERG) responses, has facilitated the identification of some of the retinal functions of Usher proteins under normal conditions and in response to challenges, such as light–dark adaptation (Lopes et al. 2011; Peng et al. 2011; Sethna et al. 2021b; Tian et al. 2016), and light exposure in albino versus pigmented genetic backgrounds (Trouillet et al. 2018). A role for myosin VIIa (Peng et al. 2011) and protocadherin-15 (Sethna et al. 2021b) in the light-induced translocation of arrestin and transducin between the inner and outer segments has been described, indicating that protocadherin-15 is essential for the rapid shuttling of proteins between photoreceptor segments. Myosin VIIa has also been implicated in opsin transport in photoreceptor-connecting cilia and the correct positioning of melanosomes in RPE cells (Liu et al. 1998a). The opsin delay in the connecting cilium (Liu et al. 1997a, 1999a), and the aberrant positioning of melanosomes and phagosomes in RPE cells observed in Myo7a-deficient mice (El-Amraoui et al. 2002; Gibbs et al. 2004; Liu et al. 1998a) also support a role for this motor protein as a cargo transporter in these cells.

For USH2, there is compelling evidence to suggest that the USH2 proteins usherin, ADGVR1, and whirlin are present in the photoreceptor periciliary ridge membrane complex (PMC) in mouse (Maerker et al. 2008; Yang et al. 2010; Zou et al. 2011), zebrafish (Dona et al. 2018) and macaque (Sahly et al. 2012) models. The PMC region is located in a spatially restricted inner segment membrane region surrounding the photoreceptor-connecting cilium (Peters et al. 1983; Watanabe et al. 1999). Like a pericuticular necklace in hair cells or a peri-terminal web region in epithelial cells, this compartment probably functions as a “hub” diffusion barrier, controlling transport to the overlying modified apical sensory cilia: the sound/motion-receptive hair bundle (Fig. 4) or the light-sensitive outer segment (Fig. 5). USH2 proteins are thought to provide structural support to the PMC region, with usherin and ADGVR1 forming fibrous links coupling the PMC to the connecting cilium (El-Amraoui and Petit 2014; Maerker et al. 2008; Mathur and Yang 2015). They are also involved in vesicle docking with the plasma membrane during protein translocation to the outer segment (Maerker et al. 2008; Toualbi et al. 2020; Zou et al. 2011).

Interestingly, a recent high-resolution imaging study with cilium- and Usher protein-specific photoreceptor markers also revealed planar polarity of the cone photoreceptors in the primate retina (Verschueren et al. 2021). The cone photoreceptors were shown to display planar polarity organized radially around the optical center of the eye, which is probably important for well-known mechanisms, such as the Stiles Crawford effect thought to align cone photoreceptors with the pupil axis through a mechanotransduction mechanism very similar to that of auditory cells (Verschueren et al. 2021). By coupling the inner segment periciliary membrane to both the cilium and the base of the outer segment (coupled to the calyceal processes in some species), the USH1 and USH2 proteins provide a dynamic and continuous cytoskeleton-based mechanical structure that is essential for the architectural integrity and function of photoreceptor cells (Sahly et al. 2012; Schietroma et al. 2017; Verschueren et al. 2021) (Fig. 5A). Further studies are required to determine the nature and extent of mechanotransduction processes within the retina (Cadoni et al. 2021), and to determine whether and how Usher proteins are involved in this process.

For USH3, no overt retinal defect was observed in *Clrn1*-deficient mice (Dulon et al. 2018; Tian et al. 2016). Potentially low levels of *CLRN1* expression in the photoreceptor cells cannot be excluded, but there is evidence for high transcript levels in the retinal inner nuclear layer of mice, pig and humans (Cosgrove and Zallocchi 2014; Dinculescu et al. 2021). The establishment of new models in non-rodent species could help to determine the key role of clarin-1 in the retina.

## Current progress in therapeutic studies on Usher syndrome

In the last decade, efficacy has been demonstrated in preclinical studies for diverse therapeutic strategies, several of which have entered clinical trials aiming to improve vision in Usher-related RP and other inherited retinal dystrophies (Botto et al. 2021; Crane et al. 2021; Sahel et al. 2019; Geleoc 2020; Toms et al. 2020b). These strategies include gene replacement, gene editing, nonsense suppression and antisense oligonucleotides (ASOs) (Botto et al. 2021; Crane et al. 2021; Sahel et al. 2019; Geleoc 2020; Toms et al. 2020b). The first studies of gene replacement therapy with a commercial adeno-associated virus (AAV) were performed on the retinas of patients with an autosomal recessive form of Leber congenital amaurosis; these patients develop severe retinal dystrophy due to biallelic mutations of *RPE65* (Maguire et al. 2019; Russell et al. 2017). Several clinical trials have been set up for treatments aiming to prolong retinal cell survival and halt vision loss in USH1 and USH2 patients. The first Usher gene therapy in humans tested in a phase I/II clinical trial involved the equine infectious anemia virus (EIAV) lentiviral-based subretinal delivery of *MYO7A* in USH1B patients (Sanofi SAR421869; Trial #NTC01505062). Another USH1B trial, also in phase I/II, is underway, for full-length myosin VIIa supplementation with a dual vector system in the retina in USH1B patients (UshTher, Horizon, 2020; Trial #NCT02065011). USH2 patients have been included in a trial of subretinal capsules of human NT-501 cells delivering ciliary neurotrophic factor (CNTF). This factor prolongs the survival of photoreceptor cells, especially cones, and halts vision loss (Trials #NCT00447980; #NCT01530659). In another phase I/II clinical trial (ProQR, Stellar; Trial #NCT03780257), USH2A patients were treated by intravitreal injection of an antisense oligonucleotide (ASO), QR-421a, designed to abolish the in-frame deletion of exon 13 of the *USH2A* gene, one of the commonest pathogenic variants of this gene in humans (Dulla et al. 2021). QR-421a has been shown to be effective in a zebrafish *Ush2a* model (Dona et al. 2018; Han et al. 2018). ASO-based targeted therapies have also been successfully used to correct a highly prevalent *USH1C* mutation, leading to significant improvements in hearing and balance functions in *USH1C* knock-in mice (Lentz et al. 2013, 2020).

Other approaches targeting specific Usher gene mutations include translational readthrough-inducing drugs (TRIDs), a new generation of aminoglycosides targeting nonsense mutations. These drugs have been assessed in the retina of *Ush1c* mice (carrying p.(Arg31*)), in which they induced harmonin expression (Goldmann et al. 2012; Nagel-Wolfrum et al. 2016). Gene editing with zinc finger nucleases and the CRISPR/Cas9 system has also been used for successful in vitro repair in stable *Ush1C* mouse cell lines carrying p.(Arg31*) and in human *USH2A* fibroblasts and induced pluripotent stem cells harboring c.2299delG p.(Glu767Serfs*21), a common *USH2A* mutation, respectively (Fuster-Garcia et al. 2017; Overlack et al. 2012) (see recent reviews Geleoc and El-Amraoui 2020; Toms et al. 2020b; Williams et al. 2017).

Patients with Usher syndrome may benefit from the fitting of hearing aids or cochlear implants, which can partially alleviate auditory sensory deprivation (for review see Davies et al. 2021). Gene replacement with AAVs of various serotypes has been successfully used to replace four Usher genes in mouse models; *USH1C* (Landegger et al. 2017; Pan et al. 2017), *USH1G* (Emptoz et al. 2017), *USH2D* (Chien et al. 2016; Isgrig et al. 2017) and *USH3A* (Dulon et al. 2018; Geng et al. 2017; Gyorgy et al. 2019). In these studies, the efficacy of hearing recovery depended on the type of USH gene, but an almost full recovery of vestibular function was obtained (for review see: Ahmed et al. 2017; Askew and Chien 2020; Delmaghani and El-Amraoui 2020; Geleoc and El-Amraoui 2020; Lustig and Akil 2019)). These findings can be explained by the low transduction efficiency of conventional AAVs in the auditory outer hair cells. Recent studies have shown that three AAVs—AAV2/9-PHPB and two synthetic AAVs, AAV2/2.m8 and AAV2/Anc80L65—efficiently transduce both inner and outer hair cells (Gyorgy et al. 2019; Isgrig et al. 2019; Landegger et al. 2017). The design of other AAVs, with optimized promoters, titers and delivery routes, is required to improve transduction rates in auditory hair cells and hearing recovery.

### Is it an Usher gene, an atypical or an ultra-rare gene?

Progress in molecular genetics, based on targeted USH exome or whole-exome sequencing methods, has greatly increased our understanding of the genetic causes and pathogenesis of Usher syndrome (Aparisi et al. 2014; Bonnet et al. 2011, 2016; Fuster-Garcia et al. 2018; Jouret et al. 2019; Stiff et al. 2020), and has revealed that some USH gene mutations can be associated with a different phenotype and Usher subtype. Recent studies have also revealed some rare cases with clinical manifestations resembling those of Usher syndrome ("Usher-like" phenotype) (Fuster-Garcia et al. 2019) (Nolen et al. 2020). The causal genes are referred to as “ultra-rare USH genes”. Below, we describe the variants of USH genes associated with atypical clinical manifestations and summarize the available data for ultra-rare USH genes.

### Usher genes: different mutations, different phenotypes

Mutations of *MYO7A* (Weil et al. 1997), *CDH23* (Bork et al. 2001), *USH1C* (Ouyang et al. 2002), *PCDH15* (Ahmed et al. 2002), *USH1G* (Maria Oonk et al. 2015), and *WHRN* (Mburu et al. 2003) can cause non-syndromic recessive hearing impairment, and *MYO7A* variants are also associated with a non-syndromic dominant form of hearing impairment (Liu et al. 1997b). The severity and progressivity of the phenotype in patients depend on the type of *MYO7A* mutation. DFNA11 patients have less severe post-lingual hearing loss than patients with DFNB2, and DFNB2 patients have variable phenotypes, ranging from profound hearing loss and variable vestibular dysfunction, or a variable age at onset for hearing loss (due to the low efficacy of expression of splicing mutations) to purely congenital profound hearing loss (Liu et al. 1997b, c; Weil et al. 1997). *USH2A* and *CLRN1* variants are associated with non-syndromic retinitis pigmentosa (Khan et al. 2011; Lenassi et al. 2015).

Several variants of the USH1 genes, *MYO7A*, *CDH23*, and *USH1G,* sometimes with digenic inheritance, have been reported to be linked to atypical USH with variable age at onset and severity of the hearing loss and RP. For instance, compound heterozygous pathogenic variants of *MYO7A* were found in two patients diagnosed with atypical USH and presenting with progressive hearing loss and mild RP (Liu et al. 1998b).

Some variants of *MYO7A* and *CDH23* have also been identified in patients with clinical diagnoses of USH2 (Aparisi et al. 2014; Bonnet et al. 2011; Fuster-Garcia et al. 2018; Zong et al. 2016), and some variants of *USH2A* have been identified in patients with clinical diagnoses of USH1 and USH3 (Aparisi et al. 2014; Bonnet et al. 2011; Fuster-Garcia et al. 2018; Zong et al. 2016). Some pathogenic variants of *USH1G* and *CDH23* have also been described in patients with atypical USH. A missense mutation of *USH1G*, c.1373A > T p.(Asp458Val), has been reported to cause atypical USH—mild retinitis pigmentosa with normal vestibular function—in two families from Turkey with autosomal recessive non-syndromic hearing loss (Kalay et al. 2005). Other frameshift mutations of *USH1G* have been reported to cause an USH1 phenotype (Weil et al. 2003; Neuhaus et al. 2017; Ouyang et al. 2005). It is, thus, possible that some missense mutations of *USH1G* result in hypomorphic alleles, leading to a less severe phenotype than for frameshift mutations. However, a biallelic frameshift mutation of *USH1G* was reported in a consanguineous Pakistani family with four affected individuals presenting moderate-to-severe hearing loss, mild retinitis pigmentosa and normal vestibular function, a phenotype similar to that of USH2 patients (Bashir et al. 2010). These studies reveal that the type or location of the mutation is not always correlated with the severity of the phenotype. An effect of modifier genes or epigenetic factors cannot be excluded.

Two USH2 genes, *USH2A* and *ADGRV1,* have also been reported to be associated with atypical USH. Mutations of *USH2A* cause USH2A, atypical USH or non-syndromic RP (Aller et al. 2004; Liu et al. 1999b; Neuhaus et al. 2017; Pierrache et al. 2016). Liu et al. reported a frameshift mutation in *USH2A* (c.2314delG/c.2299delG) in patients from 12 families, in four of which atypical USH features were observed, with progressive hearing impairment, variable vestibular function, and RP (Liu et al. 1999b). Several other mutations of this gene have also been reported to cause both USH2 and atypical USH (Aller et al. 2004; Blanco-Kelly et al. 2015; Garcia-Garcia et al. 2011; Neuhaus et al. 2017; Steele-Stallard et al. 2013). As clinical phenotypes are progressive and variable between patients with the same mutation of *USH2A*, other factors, such as age and environment (noise exposure) may account for the phenotypic variations observed in these patients.

Finally, frameshift and missense mutations of *ADGRV1* have been reported in association with typical USH2 symptoms. However, three affected siblings with previously unknown frameshift mutations were reported to have post-lingual hearing loss and an absence of night blindness, a phenotype that is different from the classical USH2 phenotype (Zhang et al. 2018).

Whatever the Usher gene, the various phenotypes observed should be considered from the angle of the causal gene identity, and the predicted impact of the causal mutation on the function of the encoded protein, which may depend on the type and location of the pathogenic variant. The position of the given gene in the Usher interactome, and the way in which its dysfunction would interfere with the functioning of other Usher proteins should also be considered, together with the possible presence of other pathogenic variants or modifier genes. Additive deleterious impacts of several variants and/or functional redundancy may account for the occurrence of a more severe, or a milder phenotype, respectively.

### Ultra-rare Ush forms or just deaf–blindness syndrome?

As mentioned above, several genes associated with Usher syndrome have been reported to result in phenotypes not consistent with the defined clinical subtypes of USH. These genes are referred to as “ultra-rare USH genes” and include *PDZD7* (Eisenberger et al. 2012), *HARS* (Puffenberger et al. 2012), *ABHD12* (Eisenberger et al. 2012), *CIB2* (Riazuddin et al. 2012), *CEP250* (Khateb et al. 2014), *CEP78* (Namburi et al. 2016), *ESPN* (Ahmed et al. 2018), and *ARSG* (Khateb et al. 2018) (see Table 1).

**Table 1:** Overview of ultra-rare USH genes reported in the literature

Gene (OMIM Ref.)	Protein product	Function	Clinical diagnosis	Variant(s)	Population	References
*PDZD7* (#612,971)	PDZD7 domain-containing 7	Scaffold protein	USH2	c.166_167insC p.(Arg56Profs*24); *USH2A* c.4338_4339del p.(Cys1447Glnfs*29) c.1750-2A > G p.(?); *USH2A* c.4515_4518 del p.(Arg1505Serfs*7);p.(Thr4439Ile) c.2194_2203del p.(Cys732Leufs*18); *ADGRV1* c.17137delG p.(Ala5713Leufs*3)	French Canadian German German	Ebermann et al. (2010)
*HARS* (#142,810)	Histidyl-tRNA synthetase	Aminoacyl-tRNA synthetase	USH3	Biallelic c.1361A > C p.(Tyr454Ser)	Plain population of Pennsylvania, Old Order Amish population of Ontario	Puffenberger et al. (2012)
			USH	c.410 G > A p.(Arg137Gln);c.262 G > A p.(Gly88Ser)	Swiss	Tiwari et al. (2016)
*ABHD12* (#613,599)	α/β-hydrolase domain-containing 12	Serine hydrolase	PHARC	Biallelic c.193C > T p.(Arg65*)	Lebanese	Eisenberger et al. (2012)
			PHARC	Biallelic c.316 + 2A > T p.(?)	Japanese	Youshimura et al. (2015)
			Atypical USH	c.316 + 5 G > A p.(?);c.477 G > A p.(Trp159*)	Chinese	Sun et al. (2018)
			Non-syndromic autosomal recessive RP	c.319delA p.(Arg107Glufs*8);c.605C > T p.(Thr202Ile)	Spanish	Nishiguchi et al. (2014)
*CIB2* (#605,564)	Calcium and integrin-binding family member 2	Calcium sensor	USH1J	Biallelic c.192G > C p.(Glu64Asp)	Pakistani	Riazuddin et al. (2012)
*CEP250* (#609,689)	Centrosomal protein 250	Centrosomal/ciliary protein	Atypical USH	Biallellic c.3463C > T p.(Arg1155*); *C2orf71* c.3289C > T p.(Gln1097*)	Iranian Jewish	Kateb et al. (2014)
			SNHL with CRD	c.361C > T p.(Arg121*);c.562C > T p.(Arg188*)	Japanese	Kubota et al. (2018)
			USH-like	c.4006C > T p.(Arg1336*);c.3337A > T p.(Lys1113*)	Spanish	Fuster-García et al. (2018)
*CEP78* (#617,110)	Centrosomal protein 78	Centrosomal/ciliary protein	SNHL with CRD	Biallelic c.893–1 G > A p.(?) Biallelic c.534delT p.(Leu179Argfs*10) c.893–1 G > A p.(?);c.534delT p.(Leu179Argfs*10)	Iranian, Iraqi, and Afghan Jewish	Namburi et al. (2016)
			SNHL with CRD	Biallelic c.499 + 1 G > T p.(?) c.499 + 5 G > A p.(?);c.633delC p.(Trp212Glyfs*18)	Greek, Swedish	Nikopoulos et al. (2016)
			Atypical USH	Biallelic c.1254 + 5 G > A p.(?) Biallelic c.1629-2A > G p.(?)	Chinese	Fu et al. (2017)
			SNHL with CRD	Biallelic c.449 T > C p.(Leu150Ser)	Belgian	Ascari et al. (2020)
*ESPN ?* (#606,351)	Espin	Actin-bundling protein	Atypical USH/ USH1M	Biallelic c2369_2386del p.(Arg790_Arg795del)	Pakistani	Ahmed et al. (2018)
*ARSG* (#610,008)	Arylsulfatase G	Lysosomal sulfatase	Atypical USH	Biallelic c.133G > T p.(Asp45Tyr)	Yemenite Jewish	Khateb et al. (2018)
				Biallelic c.130G > A p.(Asp44Asn)	Spanish	Abad-Morales et al. (2020)
				Biallelic c.1326del p.(Ser443Alafs*12) c.253 T > C p.(Ser85Pro);c.338G > A p.(Gly113Asp)	Portuguese	Peter et al. (2020)
				Biallelic c.1270C > T p.(Arg424Cys)	Persian	Fowler et al. (2021)
				c.283C > T p.(Arg95Trp);c.566 + 3_566 + 8del p.(?) c.1004C > T p.(Thr335Met);c.1326del p.(Ser443Alafs*12) Biallelic c.337G > A p.(Gly113Ser)	ND	Igelman et al. (2021)
				c.1212 + 1G > A p.(Val405Ilefs*41);c.275 T > C p.(Leu92Pro) c.1326del p.(Ser443Alafs*12);c.1024C > T p.(Arg342Trp) in combination with a heterozygous variant in *USH2A* c.6524G > A p.(Arg2175His) c.588C > A p.(Tyr196*);c.705-3940_ 982 + 2952del p.(Ser235Argfs*29) in combination with heterozygous variants in *ADGRV1* c.5525-7C > T p.(?); *MYO7A* c.905G > A p.(Arg302His); *PCDH15* c.1098 + 2354G > A p.(?)	Turkish, Caucasian	Velde et al. (2022)

*PHARC* polyneuropathy, hearing loss, ataxia, retinitis pigmentosa, and cataract, *RP* retinitis pigmentosa, *SNHL* sensorineural hearing loss, *CRD* cone–rod dystrophy, *ND* not determined

New variants in *AHBD12*, *CEP250*, and *CEP78* have been recently reported in the patients presenting with dual hearing and vision loss (see Igelman et al. 2021)

## *PDZD7* (PDZD7 domain-containing 7)

*PDZD7* encodes a PDZ scaffold protein found in the ankle-link complexes of hair cell stereocilia (Grati et al. 2012; Morgan et al. 2016; Zou et al. 2014). This gene was first identified as a possible USH gene based on of its high degree of sequence similarity to *USH1C* and *WHRN* (Schneider et al. 2009). Furthermore, in three clinical cases of USH, heterozygous pathogenic variants of *PDZD7* were identified as possible USH disease modifiers and contributors to digenic Usher syndrome (Ebermann et al. 2010). In one family with a homozygous loss-of-function (LOF) variant of *USH2A* and a monoallelic variant of *PDZD7*, the retinal phenotype was more severe. In the second family, a compound heterozygous variants in *USH2A* and a heterozygous variant in *PDZD7* caused milder retinal phenotype. In the third family, monoallelic LOF variants of *ADGRV1* and *PDZD7* led to a severe retinal phenotype (Ebermann et al. 2010) (see Table 1). However, digenic heterozygous mutant mice of *Pdzd7* with *Ush2a*, *Adgrv1* or *Whrn* did not exhibit congenital hearing impairment (Zou et al, 2014), suggesting that the digenic inheritance of two variants in two USH2 complex genes reported in these families was most likely coincidence rather than causal. In the cochlea, PDZD7 preferentially interacts with ADGRV1 rather than with USH2A (Chen et al. 2014). Moreover, PDZD7 defects have been shown to affect the localization of the USH2 protein complex in cochlear hair cells, but not in photoreceptors (Zou et al. 2014; Chen et al. 2014; Du et al. 2018). Several biallelic mutations of *PDZD7* have been found to cause non-syndromic recessive mild-to-severe sensorineural hearing loss (Booth et al. 2015; Vona et al. 2016; Guan et al. 2018; Luo et al. 2019; Lee et al. 2019). Interestingly, two Chinese patients with the same variant reported in Ebermann study, c.166_167insC p.(Arg56Profs*24), presented normal retinal and vestibular phenotypes (Guan et al. 2018). The observed divergent phenotypes in former patients may be associated with *USH2A* variants (Ebermann et al. 2010). The knockdown of *Pdzd7* function in zebrafish results in an USH-like phenotype, manifesting as a reduced startle reflex, circling, and disorganized bundles of stereocilia in the cochlea, together with progressive retinal cell death, with a decrease in the amount of ADGRV1 localizing to the connecting cilia in photoreceptor cells. In contrast, Ush2a levels and localization appeared normal in *Pdzd7a* knockdown retinas (Ebermann et al. 2010). Moreover, *Pdzd*7-knockout mice display profound hearing loss and a disorganization of the ankle-link region of the stereocilia in hair bundles, together with weak mechanoelectrical transduction currents and low cochlear hair cell sensitivity (Zou et al. 2014). It has been suggested that, by interacting with both USH1 and USH2 proteins, including myosin VIIa, usherin, ADGRV1 and whirlin, PDZD7 forms part of the USH2 interactome (Du et al. 2018) (see Fig. 3D). PDZD7 can function as a modifier gene for USH, but additional data are required to confirm whether mutations of *PDZD7* alone are sufficient to cause Usher syndrome.

## *HARS* (histidyl-tRNA synthetase)

*HARS* encodes histidyl-tRNA synthetase (HARS), a homodimeric class IIa aminoacyl tRNA synthetase that charges tRNA molecules with histidine amino acids for protein translation (Antonellis and Green 2008). In 2012, *HARS* was reported to be a causal gene for the USH3 phenotype based on a homozygous pathogenic variant found in two patients with hearing and vision impairments (Puffenberger et al. 2012). Compound heterozygous pathogenic variants of *HARS* were subsequently described in a patient with an undefined type of USH (Tiwari et al. 2016). The subcellular distribution of HARS in the inner ear and retina remains to be determined and it is unknown whether this protein is part of the USH protein network. In addition to typical USH3 symptoms, *HARS*-affected patients displayed signs of episodic psychosis and neurological symptoms (Puffenberger et al. 2012). The presence of additional symptoms is *HARS*-affected patients raises the question as to whether *HARS* is a genuine USH-causing gene.

## *ABHD12* (α/β-hydrolase domain-containing 12)

*ABHD12* (α/β-hydrolase domain-containing 12) encodes ABHD12, a serine hydrolase that hydrolyzes 2-arachidonoyl glycerol, an endocannabinoid lipid transmitter that acts on the cannabinoid receptors CB1 and CB2 (Eisenberger et al. 2012; Li et al. 2019; Sun et al. 2018). Mutations of *ABHD12* have been reported in PHARC syndrome, which is characterized by a combination of polyneuropathy, hearing loss, ataxia, retinitis pigmentosa, and early-onset cataracts. However, several biallelic pathogenic variants have been identified in patients clinically diagnosed with USH3-like symptoms or an autosomal recessive non-syndromic form of retinal degeneration (Eisenberger et al. 2012; Nishiguchi et al. 2014; Sun et al. 2018; Yoshimura et al. 2015). One major problem with these studies is that they do not usually include neurological results for the patients, potentially leading to the misdiagnosis of their condition as USH3. In addition, *Abhd12*-knockout mice display neurodegenerative abnormalities resembling the behavioral phenotypes of human PHARC patients (Blankman et al. 2013). PHARC should, therefore, be considered as a differential diagnosis for patients with USH3-like phenotypes presenting with progressive hearing loss and balance defects. As for *HARS*, further studies are required to determine the subcellular distribution of ABHD12 in the inner ear and retina, and its interactions with other USH proteins (Fig. 3D), to confirm that there is a genuine association with Usher syndrome.

## *CIB2* (calcium and integrin-binding family member 2)

*CIB2* encodes an EF domain-containing protein that binds both calcium and integrin. Biallelic mutations of this gene were initially reported to cause non-syndromic recessive deafness (DFNB48) and Usher syndrome (USH1J) (Riazuddin et al. 2012). Only one family with USH1J carrying a *CIB2* mutation has been identified to date (Riazuddin et al. 2012). *CIB2* is widely transcribed in many tissues, including the inner ear and retina. It is expressed in the stereocilia of auditory hair cells and in the retinal photoreceptor and pigmented epithelium cells (Riazuddin et al. 2012). CIB2 has been shown to interact with two components of the Usher interactome: myosin VIIa (USH1B) and whirlin (USH2D) (Riazuddin et al. 2012) (see Fig. 3D). Sequencing studies on 427 patients with USH led to the identification of biallelic or monoallelic mutations of Usher genes in 421 individuals with USH, but no *CIB2* mutations were detected in any of these patients (Bonnet et al. 2016). In recent years, several pathogenic variants of *CIB2* were identified after the discovery of USH1J, in families with the DFNB48 form of isolated deafness but no signs of retinitis pigmentosa or vestibular dysfunction (Patel et al. 2015; Seco et al. 2016; Michel et al. 2017; Booth et al. 2018; Souissi et al. 2021). Morphofunctional analyses of the inner ear in *Cib2*^−/−^ mice showed that a lack of *Cib2* leads to abnormal auditory hair bundle shape and an absence of mechanoelectrical transduction responses in cochlear hair cells (Giese et al. 2017; Liang et al. 2021; Michel et al. 2017; Wang et al. 2017). Unlike mice defective for any of the five unequivocal USH1 genes, *Cib2*^−/−^ mice have no vestibular defect (Giese et al. 2017; Michel et al. 2017; Wang et al. 2017). However, a thorough longitudinal study in conditional *Cib2*^flox/flox^; RPE-*Cre* + mice showed that specific inactivation of *Cib2* in the subretinal pigment epithelium (RPE) cells led to age-related retinal phenotypes (Sethna et al. 2021a). It has been suggested that *Cib2* loss leads to dysregulated mTORC1 signaling and a decrease in autophagy, resulting in RPE cells dysfunction and secondary photoreceptor dysfunction (Sethna et al. 2021a). These abnormalities trigger an age-related decline of retinal functions manifesting as RPE deposits, a marked accumulation of drusen (extracellular lesions characteristic of dry age-related macular degeneration) and impaired visual function (Sethna et al. 2021a). Caution is, therefore, required when providing genetic counseling for patients carrying *CIB2* mutations. These mutations may not lead to Usher syndrome, but the possibility of some types of *CIB2* mutations conferring predisposition to age-related macular degeneration should be considered.

## *CEP250* (centrosomal protein 250)

*CEP250* encodes ciliary protein C-NAP1, a member of the CEP family (Kumar et al. 2013). This protein family has more than 30 members, which form the core component of the centrosome and play crucial roles in centriole biogenesis, centrosome cohesion, and the control of cell-cycle progression (Kumar et al. 2013). *CEP250* is expressed in the photoreceptor outer segment and involved in retinal ciliogenesis (de Castro-Miro et al. 2016). In 2014, Khateb et al. identified a homozygous pathogenic variant of *CEP50* in a family affected by an USH-like condition, with mild RP and early-onset hearing loss (Khateb et al. 2014). However, the patients in this family also carried a monoallelic or biallelic mutation of *C2orf71*, encoding a ciliary protein associated with autosomal recessive RP. Patients double-homozygous for the mutations of both genes had a more severe phenotype, indicating an additive effect of the dysfunctions of these two ciliary proteins due to nonsense mutations (Khateb et al. 2014). Furthermore, compound heterozygous mutations of *CEP250* have been reported in patients, manifesting as mild cone–rod dystrophy and neurosensorial hearing loss (Kubota et al. 2018). Two heterozygous nonsense mutations of *CEP250* have also been reported in a patient presenting with an Usher-like phenotype (Fuster-Garcia et al. 2018). However, progressive hearing loss in this patient was associated with late-onset visual impairment due to cone–rod dystrophy (Fuster-Garcia et al. 2018), contrasting with the typical rod–cone dystrophy of USH patients. A nonsense mutation of *CEP250* has been reported in a consanguineous family with retinitis pigmentosa with no hearing or vestibular impairment (Huang et al. 2019). The study of *Cep250-*knock-in mice harboring the same mutation showed that a lack of Cep250 leads to severe retinal degeneration. Cep250 defects affect cilium and protein localization in the outer segments of photoreceptor cells, significantly decreasing retinal thickness and ERG responses. This finding confirms the crucial role of CEP250 in photoreceptor cilium development and function (Huang et al. 2019), but the potential function of CEP250 in the inner ear and its possible interactions with other proteins of the Usher network remain unknown (Fig. 3D).

## *CEP78* (centrosomal protein 78)

*CEP78* is a ciliary gene encoding CEP78, another member of the CEP family of proteins localizing to the centriole wall. Its interaction with the N-terminal catalytic domain of PLK4, a serine/threonine polo-like kinase, is important for PLK4-induced centrosome overduplication and cell-cycle regulation (Brunk et al. 2016). CEP78 is found in the photoreceptor-connecting cilium, with a stronger signal in cones than in rods (Namburi et al. 2016; Nikopoulos et al. 2016). The *CEP78* transcript is also present in the human cochlea (Nikopoulos et al. 2016). As for *CEP250*, several biallelic pathogenic variants of *CEP78* have been reported in patients presenting with progressive and late-onset cone–rod degeneration and hearing loss (Fu et al. 2017; Namburi et al. 2016; Nikopoulos et al. 2016). However, pathogenic variants of CEP78 may be associated with phenotypes other than typical USH-like phenotypes. For instance, a homozygous *CEP78*-truncating variant was identified in a family diagnosed with autosomal recessive non-syndromic RP (de Castro-Miro et al. 2016). Ascari et al (2020) identified a missense mutation of *CEP78* in the homozygous and compound heterozygous states in three unrelated families displaying cone–rod retinal degeneration and hearing loss, with and without male infertility (Ascari et al. 2020). Given the complete characterization of the clinical phenotype in these studies, *CEP78-* and *CEP250-*affected patients can be classified as a distinct group with a “cone–rod degeneration and sensorineural hearing loss” syndrome rather than an Usher-like condition.

## *ESPN* (espin)

*ESPN* encodes espin, a calcium-insensitive actin-bundling protein essential for hearing and vestibular function. A frameshift mutation affecting the espin C-terminal actin-bundling module causes deafness and vestibular behavioral defects accompanied by stereocilium shortening and disorganization in the jerker mouse (Zheng et al. 2000). Espin is localized in the stereocilia of auditory and vestibular hair cells (Sekerkova et al. 2006; Zheng et al. 2000) and in the photoreceptor calyceal processes (Sahly et al. 1997), where it plays an essential role in actin filament crosslinking to control the length and integrity of microvillar apical membranes. Furthermore, an in-frame deletion of *ESPN* was reported to underlie atypical USH1 in a Pakistani family (Ahmed et al. 2018). All the patients from this family had hearing loss, vestibulopathy, progressive retinal abnormalities and vision impairment, including temporal flecks (delayed dark adaptation with an irregular retinal contour), optic disk pallor and weak scotopic ERG responses (Ahmed et al. 2018). Espin has been shown to interact with whirlin, and its expression is altered in the photoreceptors and hair cells of whirlin-deficient mice (Wang et al. 2012), linking this protein to the hair bundle-based Usher interactome (see Fig. 3D). However, unlike the loss-of-function mutations of other USH1 genes, biallelic frameshift mutations of *ESPN* in humans cause only non-syndromic prelingual hearing loss (DFNB36) and vestibular areflexia without vision impairment (Naz et al. 2004). Several pathogenic variants have also been reported in patients with autosomal dominant progressive hearing loss without vestibular or vision deficits (Donaudy et al. 2006). Additional information is required about the pathogenic impact of the in-frame *ESPN* mutation, which is considered to be an atypical Usher variant, and whether it is causal for all the clinical symptoms observed in *ESPN*-affected patients (Ahmed et al. 2018). On fundoscopy, patients of about 30 years of age with *ESPN* mutations do not display the typical RP observed in age-matched USH1 patients (Ahmed et al. 2018). The preserved normal cone function in the retina of an affected 42-year-old deaf individual, and the occurrence of cardiac abnormalities in other affected patients remain unexplained (Ahmed et al. 2018). The type of mutation may account for variability in the phenotype of patients in these studies, but the possible effects of another pathogenic variant causing retinal impairment, age, and environmental factors should be considered.

## *ARSG* (arylsulfatase G)

*ARSG* encodes arylsulfatase G, a lysosomal sulfatase widely expressed in various human tissues and involved in diverse metabolic pathways (Ferrante et al., 2002). *Arsg* deficiency in a knockout mouse model of mucopolysaccharidosis IIIE results in ataxia, a progressive degeneration of rod photoreceptor cells accompanied by reactive astrogliosis and microgliosis, and a dysregulation of several lysosomal proteins (Kowalewski et al. 2015). A homozygous founder variant of *ARSG* has been reported in patients from three families of Yemenite Jewish origin suffering from an atypical form of late-onset hearing and vision impairments, referred to as atypical USH (Khateb et al. 2018). The patients displayed moderate-to-severe sensorineural hearing loss and a retinal degeneration phenotype, with ring-shaped atrophy along the arcades surrounding the fovea, resulting in ring scotoma (Khateb et al. 2018). Furthermore, several additional pathogenic variants have been reported to be associated with this deaf–blindness syndrome (see Table 1) (Abad-Morales et al. 2020; Fowler et al. 2021; Igelman et al. 2021; Khateb et al. 2018; Peter et al. 2021; Velde et al. 2022), All *ARSG*-affected patients were diagnosed with RP with a midlife age of onset (35–60 years) (Abad-Morales et al. 2020; Fowler et al. 2021; Igelman et al. 2021; Khateb et al. 2018; Peter et al. 2021; Velde et al. 2022), which is later than generally seen in classical Usher forms. Also, the *ARSG* late-onset RP is predominantly pericentral, accompanied by macular changes (Abad-Morales et al. 2020; Fowler et al. 2021; Igelman et al. 2021; Khateb et al. 2018; Peter et al. 2021; Velde et al. 2022), distinct from the typical Usher rod–cone dystrophy. As for hearing loss, all but one previously described individuals (Igelman et al. 2021) display progressive mild, moderate-to-severe hearing loss with a self-reported onset ranging from childhood to 50 years (Abad-Morales et al. 2020; Fowler et al. 2021; Igelman et al. 2021; Khateb et al. 2018; Peter et al. 2021; Velde et al. 2022). Unlike USH patients that benefit from cochlear implants (Davies et al. 2021), *ARSG*-affected individuals rely on hearing aids, which starts at varying ages ranging from 18 to 67 years (Abad-Morales et al. 2020; Fowler et al. 2021; Igelman et al. 2021; Khateb et al. 2018; Peter et al. 2021; Velde et al. 2022). The dual occurrence of late-onset hearing impairment and vision loss in *ARSG* phenotype is consistent among patients, confirming that *ARSG* LOF variants do lead to a deaf–blindness syndrome. So far, there is no evidence of *ARSG* association with non-syndromic vision or hearing loss. The unique RP and hearing phenotypic features due to *ARSG* LOF variants (Abad-Morales et al. 2020; Fowler et al. 2021; Igelman et al. 2021; Khateb et al. 2018; Peter et al. 2021; Velde et al. 2022) and lack of connection with Usher proteins (see Fig. 3D) make the designation as Usher type IV unnecessary. Unlike the phenotype in the knockout mice (Kowalewski et al. 2015; Kruszewski et al. 2016) and *ARSG*-deficient dogs (Abitbol et al. 2010), none of *ARSG*-affected patients show any signs of lysosomal storage defects in peripheral organs or central nervous system involvement (Abad-Morales et al. 2020; Fowler et al. 2021; Igelman et al. 2021; Khateb et al. 2018; Peter et al. 2021; Velde et al. 2022). The precise function of ARSG in the retina and the inner ear remain to be established. Also, attention should also be paid to occurrence of other phenotypes, such as a medical history of hypothyroidism observed in two *ARSG*-affected patients (Velde et al. 2022).

In conclusion, to clarify the diagnosis of Usher syndrome, there is growing evidence that strict adherence to the clinical criteria for Usher syndrome is essential for the correct diagnosis of this condition. In addition to clinical information about hearing and vision, detailed information about vestibular function is required for a patient to be considered to have USH1. The presence of retinal degeneration classified as a rod–cone dystrophy is also a key criterion for the diagnosis of Usher syndrome. Attention should be paid to the presence of symptoms affecting additional organ systems, which could be signs that the disease falls within other syndromic deaf–blindness disorders. Once a defective gene is identified, available or predictive information about the function of the defective protein in the inner ear and the eye, in humans or animal models (cf. below), and its potential interactions with known Usher proteins should guide the decision as to whether the gene can be considered or maintained as an Usher gene.

## Conclusion and proposal of a new, revised classification for Usher syndrome

With growing access to advanced genomic sequencing, a wealth of additional pathogenic variants of known USH genes (or other genes) are being identified in patients with hearing and vision loss (Bonnet et al. 2016; Lenarduzzi et al. 2019; Bolz 2020; Nolen et al. 2020; Stiff et al. 2020; Nisenbaum et al. 2021; Castiglione and Moller 2022). The previous three-category classification was based exclusively on clinical criteria, but the co-occurrence of hearing and vision deficits, of any type, should not be the only criterion for the diagnosis of Usher syndrome. We agree with several other researchers and clinicians (Bolz 2020; Nolen et al. 2020; Stiff et al. 2020; Nisenbaum et al. 2021) that the time has come to revisit and complement the traditional Usher syndrome classification.

The use of all available criteria and data on disease-causing genes should make it possible to establish clear diagnoses without blurring the boundaries between Usher and non-Usher forms of deaf–blindness, and this will be of benefit to the clinicians, scientists, and patients. With the advent of precision medicine, all the available information on clinical symptoms, genotype–phenotype correlations, interactions within the Usher molecular network, and precise pathogenic events in animal models could be considered to determine whether pathogenic variants should be considered causal for Usher syndrome. Based on clinical, genetic, network, and disease model findings for the founding forms of Usher syndrome, USH1, USH2 and USH3 (see Figs. 4, 5), and those for additional so-called ‘USH-like’ genes, we propose to consider *MYO7A, USH1C, PCDH15, CDH23*, and *USH1G* as the only USH1 genes, *USH2A, ADGRV1,* and *WHRN* as the only USH2 genes, and *CLRN1* as the causal gene for USH3. Depending on the type and location of the causal variant, it is not uncommon to observe variations of clinical expression in rare diseases. The variety of phenotypic expression in patients carrying USH gene mutations should, therefore, be explained and accepted within the spectrum of Usher syndrome; the inference of atypical Usher forms is unnecessary. New guidelines are probably needed to establish clear consensus criteria for the diagnosis of USH and the inclusion of new genes as actual “USH” genes. *PDZD7* may act as a modifier gene for USH, but *HARS*, *ABHD12*, *CIB2*, *CEP250, CEP78*, *ESPN*, and *ARSG* could be grouped in a separate syndromic “deaf–blindness” category, to avoid diagnostic pitfalls and misinformation during genetic counseling for Usher syndrome. Providing an early and accurate diagnosis is essential for Usher syndrome, to ensure appropriate patient management choices for personalized treatment and interventions. Particularly for patients with USH1, the most severe form, early diagnosis makes it possible to fit cochlear implants to alleviate the burden of hearing loss, and to start monitoring for retinitis pigmentosa early, as the progression of this condition would affect the performance of the implant if the ability to lip-read were lost. Detailed clinical investigations should be performed for hearing, balance, and vision, to establish optimal genotype–phenotype correlations, not only according to the identity of the causal gene but also taking the nature and severity of the mutation into account. Careful attention should also be paid to the presence of “mono or biallelic” additional mutations in potentially co-segregating genes that may account for the variable spectrum of clinical symptoms. The accumulation of data over time might help to establish predictive models for the spectrum of disease symptoms, their progression and severity. Recent progress towards elucidating the mechanisms of Usher disease is fueling an intensification of efforts to develop various therapeutic strategies and to establish clinical trials for Usher syndrome, with approaches including gene replacement, gene editing, antisense oligonucleotides, and small-molecule drugs (Williams et al. 2017; Geleoc and El-Amraoui 2020; Delmaghani and El-Amraoui 2020; Toms et al. 2020b; Botto et al. 2021; Dinculescu et al. 2021). Success for these promising therapies, the clarity of diagnosis, and the dissemination of more precise messages to researchers, clinicians, ENT specialists, ophthalmologists and all those working in sensory fields will be transformative, fostering the rapid transfer of today’s promises into future clinical applications for patients.

## Data Availability

Not applicable.
